# Morphology and Viscoelasticity of Actin Networks Formed with the Mutually Interacting Crosslinkers: Palladin and Alpha-actinin

**DOI:** 10.1371/journal.pone.0042773

**Published:** 2012-08-16

**Authors:** Brian Grooman, Ikuko Fujiwara, Carol Otey, Arpita Upadhyaya

**Affiliations:** 1 Department of Physics, University of Maryland, College Park, Maryland, United States of America; 2 National Heart Lung and Blood Institute, National Institutes of Health, Bethesda, Maryland, United States of America; 3 Department of Cell and Molecular Physiology, University of North Carolina, Chapel Hill, North Carolina, United States of America; 4 Department of Physics and Institute for Physical Science and Technology (IPST), University of Maryland, College Park, Maryland, United States of America; Dalhousie University, Canada

## Abstract

Actin filaments and associated actin binding proteins play an essential role in governing the mechanical properties of eukaryotic cells. Even though cells have multiple actin binding proteins (ABPs) that exist simultaneously to maintain the structural and mechanical integrity of the cellular cytoskeleton, how these proteins work together to determine the properties of actin networks is not clearly understood. The ABP, palladin, is essential for the maintenance of cell morphology and the regulation of cell movement. Palladin coexists with 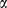-actinin in stress fibers and focal adhesions and binds to both actin and 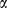-actinin. To obtain insight into how mutually interacting actin crosslinking proteins modulate the properties of actin networks, we characterized the micro-structure and mechanics of actin networks crosslinked with palladin and 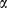-actinin. We first showed that palladin crosslinks actin filaments into bundled networks which are viscoelastic in nature. Our studies also showed that composite networks of 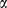-actinin/palladin/actin behave very similar to pure palladin or pure 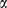-actinin networks. However, we found evidence that palladin and 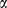-actinin synergistically modify network viscoelasticity. To our knowledge, this is the first quantitative characterization of the physical properties of actin networks crosslinked with two mutually interacting crosslinkers.

## Introduction

Cells carry out a multitude of functions through the control and rearrangement of a structural network called the cytoskeleton, which is comprised of biopolymer filaments and numerous other proteins [1]. Actin filaments are key structural and mechanical components of the cytoskeleton. They are crosslinked by actin binding proteins into higher order structures of meshes, bundles or composite bundled networks [2], [3]. The actin cytoskeleton imparts mechanical integrity to cells enabling them to generate or respond to forces, an ability critical for proper embryonic development, wound healing, cell movement and tissue homeostasis [4]–[6]. In order to carry out these diverse functions, cells dynamically regulate their cytoskeletal structures altering their local mechanical properties. Studying the mechanical properties of cytoskeletal networks that arise from the interaction between actin filaments and ABPs is, therefore, of utmost importance for furthering our understanding of cellular mechanics.

Palladin is a recently described protein that is ubiquitous in mammals, with multiple isoforms expressed in a tissue-specific manner [7]. Palladin is found in lamellar actin networks and stress fibers, structures that are critical for cell movement and sensing of the mechanical environment [7]. Two immunoglobulin-like domains (Ig3 and Ig4) appear to bind f-actin with an apparent dissociation constant, 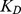, of 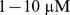, and full length palladin has been shown to bundle actin networks *in vitro* at very high concentrations [8]. Changes in the level of palladin expression causes striking alterations in the morphology of the actin cytoskeleton leading to defects in cell shape and movement [9]–[12]. Palladin knockout in mice is lethal at mid-gestation, with profound defects stemming from aberrant cell motility during development [13], [14]. This phenotype clearly demonstrates that other actin crosslinking proteins are not able to substitute for palladin or compensate for its loss of expression during embryonic development. However, despite the critical importance of palladin/actin interaction, the influence of palladin on the structural and viscoelastic properties of actin networks is not well understood. Palladin also binds 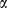-actinin [7], a prominent actin-binding protein, and these two proteins colocalize in stress fibers and focal adhesions [15]. While the viscoelastic properties of actin/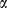-actinin networks have been well studied [16]–[20], it is not known whether palladin modifies the structure and mechanical behavior of networks crosslinked with 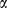-actinin.

As the *in vivo* cytoskeleton is extremely complex, elucidating the basic principles governing cytoskeletal mechanics in cells is difficult. *In vitro*, studies where crosslinker composition and properties can be precisely controlled, are advantageous for the study of the mechanical behavior of actin networks [21], [22]. Actin monomers assemble *in vitro* into filamentous networks that behave like weak viscoelastic solids [23], which stiffen in the presence of crosslinkers [16], [19], [20], [24]–[31]. These networks exhibit remarkable material properties owing to the semi-flexible nature of the actin filaments as well as the structure, affinity and compliance of the individual crosslinkers [32]. Despite advances in the study of crosslinked actin networks, the physical principles which lead to the formation of more complex structural arrangements, such as filament bundles and networks of bundles are not well understood [29], [33]–[35]. Further, most studies so far have focused on studying the mechanical properties of actin networks crosslinked with a single actin crosslinker. However, in cells, several actin crosslinking proteins co-exist in the same subcellular region. The heterogeneous cytoskeletal structures seen in cells arise in part due to the simultaneous presence of multiple crosslinkers, each imparting a particular structure and mechanical character to the network in isolation. There have been a few *in vitro* studies involving multiple crosslinkers [30], [31], but it remains unclear as to whether they function synergistically or independently to alter the viscoelastic properties of the networks [16].

Our motivation for this work was two-fold: 1) to characterize the structural and viscoelastic properties of actin networks crosslinked by palladin and 2) to study whether palladin modifies the network morphology and viscoelasticity of actin networks crosslinked by 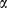-actinin. We find that palladin induces the formation of bundled actin networks as evidenced by network morphology and rheology. Increasing palladin concentrations led to changes in morphology of the network resulting in an enhancement of the linear network stiffness. We also found that 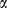-actinin and palladin do not behave independently in modulating the mechanical properties of composite actin networks.

## Results

### Palladin crosslinks actin into bundled networks

**Figure 1:**
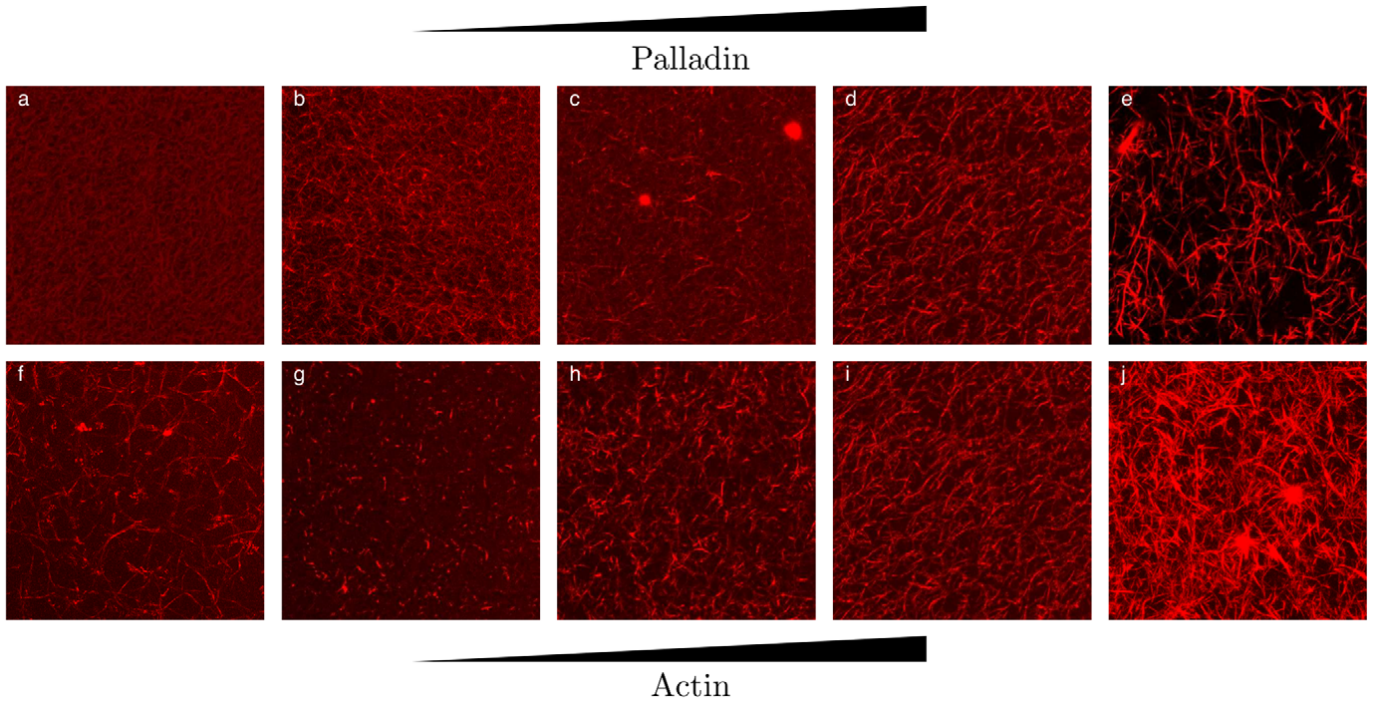
Palladin forms bundled actin networks. (a)–(e): Maximum intensity projections of confocal stacks of actin networks (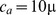
M) with palladin 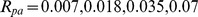 and 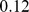 respectively. (f)–(j): Maximum intensity projections of confocal stacks of actin networks with fixed 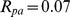 and varying actin concentrations (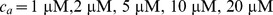 respectively). Images are 72 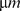 per side.

We allowed actin filaments to polymerize in the presence of palladin and crosslink into networks and imaged them on a confocal microscope. We observed distinct morphological changes of the actin network depending on both the concentration of actin (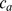) and the actin-palladin ratio, denoted by 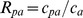. Maximum intensity projections of fluorescent confocal images of networks with fixed 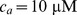 and varying 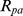 are shown in Figure 1. For very low concentrations of crosslinker (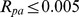) the networks were visually indistinguishable from entangled f-actin networks (Fig. 1a). As the concentration of crosslinkers was increased (above 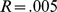), small bundles become apparent within the entangled f-actin network. The initial appearance of bundles was characterized by continuous groups of pixels with intensities greater than 1.5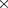 the average. The depletion of f-actin in the bundled phase appeared as a change in the contrast of the image (80–90% of the dynamic range of the sensor for bundles versus 20% or below for entangled f-actin). Further increase in palladin concentration yielded networks with thicker bundles and larger spaces between bundles as indicated by a stronger depletion of f-actin in the dark regions between bundles and brighter fluorescence in the bundled regions (Fig. 1b–c). At higher crosslinker concentrations, above 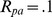, the bundles formed dense clusters (Fig. 1d), which seemed to be separated from each other and the network no longer appeared continuous across the sample.

We next examined the effect of actin concentration on actin/palladin networks. We fixed the palladin ratio, 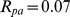, a concentration regime where palladin forms bundled homogenous networks well above the bundling threshold, and varied the actin concentration from 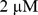 to 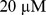 (Fig. 1e–h). At this ratio, filaments formed bundled networks that were initially sparse for low 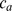 and formed spanning networks with larger voids for increasing 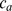. At a very high 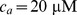, we found that palladin appeared to bundle actin extremely efficiently, forming numerous overlapping bundles as well as clusters where the bundle concentration is very high. Our observations indicated that above a critical concentration, palladin organized actin filaments into a crosslinked network of branched bundles which were slightly curved. Similar structural changes have also been observed in actin networks grown in the presence of filamin [29]. Actin filaments cross-linked by fascin form tight straight bundles while in the presence of filamin, they form long curved bundles and networks of branched bundles [30].

It is known that macroscopic network elasticity is linked to microscopic network structure which can depend sensitively on the details of the interaction of crosslinkers with actin filaments [18]. A useful quantity to measure in a network of filaments is the mesh size or pore size which captures the characteristic distance between bundles of filaments in 3D or effectively the size of the spaces between bundles that are devoid of f-actin. Previous studies of crosslinked actin networks have characterized the network structure qualitatively using visual depiction of the types of bundles formed, or measured the average spacing between bundles from an estimate of peak-to-peak distance between bundles across one cross-section of the sample, thereby underestimating the pore size [18]. While there have been some studies to quantify collagen and other filamentous networks [36]–[38], to our knowledge, the mesh size of cross-linked actin networks has not been carefully characterized as a function of crosslinker concentration.

**Figure 2:**
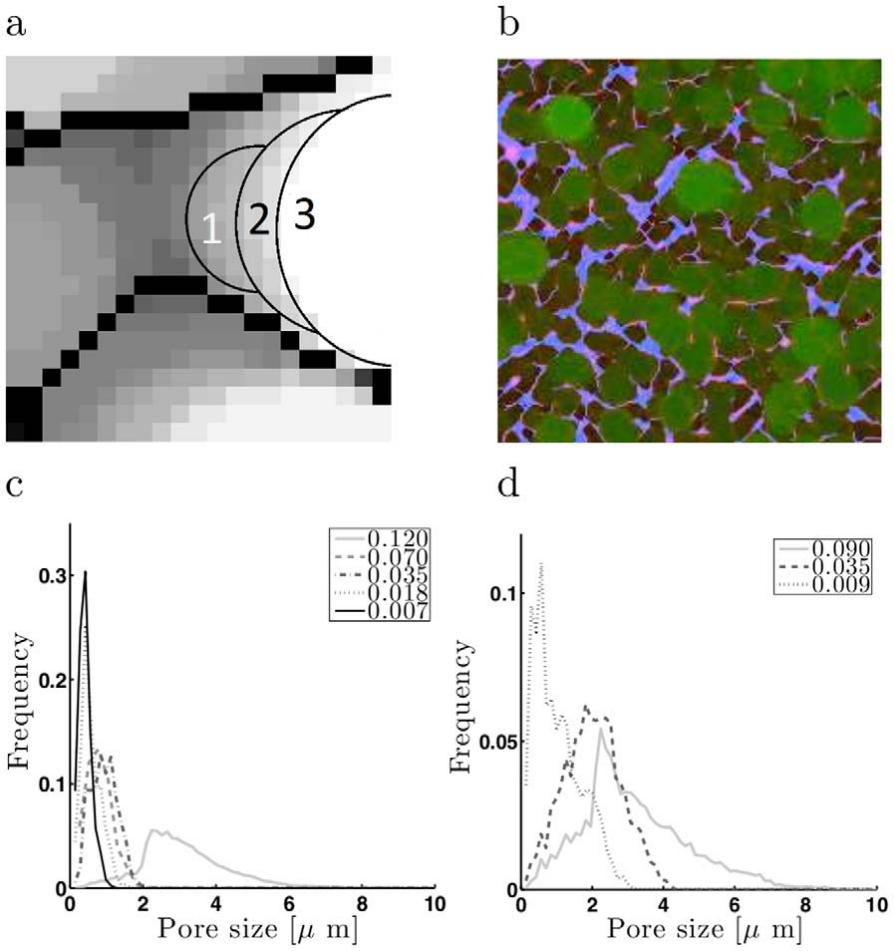
Pore-size analysis of actin networks. (a) Close-up illustration of the covering spheres method. Black pixels represent actin bundles, gray and white pixels represent covering spheres of three different radii. The thin black lines show edges of representative spheres. Here, sphere 1 is partially covered by a larger sphere 2, and sphere 2 is partially covered by sphere 3. The voxels enclosed by a sphere, but left uncovered by a larger sphere, are those counted for the pore size analysis. For example, the 19 voxels whose centers are in the crescent shaped region (2) would contribute to the total for pores of 12 pixel diameters because that is the diameter of sphere 2, and it is the largest such sphere which covers those voxels. (b) An example of the resulting pore size analysis using the covering spheres method. Red pixels are the original image, light blue pixels are a binary representation of the actin bundles. Green pixels are pixels from the covering spheres; the brighter the green the larger the pore size. Image is 29 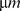 along each side. (c) Pore size distribution of 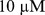 actin networks for different 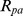. (d) Pore size distribution for 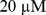 actin networks for different 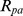. Note that the higher concentration of crosslinkers leads to larger pore sizes.

We adapted a method based on a covering sphere algorithm to quantify the microstructure of actin networks from confocal image stacks (Fig. 2a,b; see Methods). This analysis is most useful for concentrations where there is a clear distinction between individual bundles and the background (Fig. 1b–d). We show the results of the analysis for 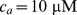 in Figure 2c. We found that networks with crosslinker concentrations below 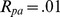 have pore-size distributions indistinguishable from pure f-actin networks. On the other hand, very high palladin concentrations, above 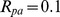, resulted in clusters of filament bundles several tens of microns across. The clusters were isolated from each other and no longer formed part of a continuous network of bundles throughout the sample. We found that with increasing palladin concentrations, 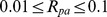, the peak of the pore size distribution shifted towards larger pore sizes. This implies that bundles get thicker as the spaces between bundles increase, and consequently the pore sizes become larger. Additionally, the pore size distribution also broadened to exhibit a prominent tail of large pores. For a higher actin concentration, 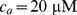, the pore size analysis showed a similar trend, with larger 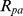 leading to larger pore sizes (Fig. 2c). Under these conditions the overall pore sizes were larger than for 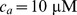 at similar palladin ratios. Higher actin concentration also led to a broader tail of the distribution, arising from stronger bundling by palladin and appearance of bundle clusters. Overall, we found that the average pore size scaled as 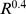 for both actin concentrations. Our observations suggest that high palladin concentrations might lead to a cooperative effect in which the presence of smaller bundles facilitates the formation of further bundles, thereby increasing the preponderance of large pores. This effect was enhanced at larger actin concentrations (Fig. 2d.) Actin/fascin networks show qualitatively similar behavior with larger crosslinker concentrations leading to an increase in the bundled phase [29]; actin/scruin networks show an increase of apparent pore size with concentration 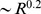 [27]. Such pore size distributions might be characteristic of networks of bundled actin filaments.

### Palladin modifies viscoelastic properties of actin networks

**Figure 3:**
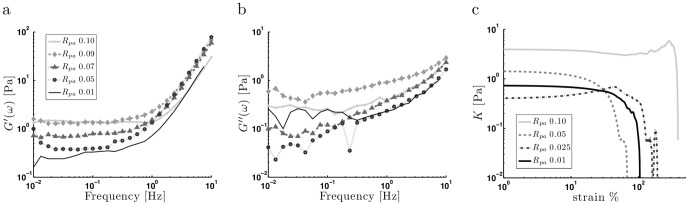
Viscoelastic properties of actin/palladin networks. (a) Storage moduli and (b) loss moduli of 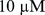 actin networks crosslinked with different concentrations of palladin. The legend is same for (a) and (b). The storage modulus is weakly sensitive to frequency at low frequencies and changes around 1 Hz to a power law relation, 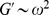. Above 1 Hz the loss modulus tends towards 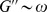. (c) Differential moduli of 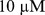 actin networks. No significant non-linear behavior is observed in these networks for any palladin concentrations.

Our next goal was to establish whether the observed structural transitions entailed significant changes in the viscoelastic properties of actin/palladin networks. Typically, cytoskeletal polymer networks are viscoelastic, characterized by an elastic modulus 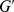 (the propensity of the polymers to rebound after shear deformation) and a viscous modulus, 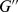 (the extent of flow under shear stress). In general, 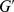 and 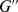 are frequency-dependent measurements indicating how materials behave solid-like at certain frequencies while behaving liquid-like at different frequencies. We characterized the viscoelastic properties of actin/palladin networks in the linear elastic limit by making rheological measurements at small deformations. We fixed the actin concentration at 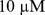 to ensure that the samples were homogeneous for all the 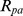 tested. We found that networks crosslinked with palladin indeed show viscoelasticity, as 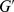 was about an order of magnitude larger than 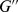 across the entire frequency range and for all concentrations tested. The frequency response of 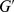 and 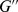 (measured at 1% amplitude strain) exhibited similar behavior for all conditions tested (Fig. 3a–b). The storage modulus was very weakly dependent on frequency from 0.01 to 1 Hz, suggesting the existence of a solid-like cross-linked gel. Above 1 Hz, 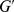 increased with frequency, showing a power law relation where 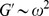 (Fig. 3a). We found that frequency response for 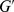 and 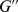 was the same across several frequency sweeps. This indicates that polymerization and cross-linking was completed and that the network had reached a stable state and the sample did not suffer damage under applied strain.

We found that the overall stiffness of the networks increased with increasing crosslinker concentration, with the plateau modulus, defined by 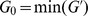 over 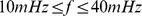, varying with concentration as 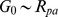. Such enhancement of network stiffness with crosslinker concentration has been observed for other crosslinkers with similar power-law dependence. The effect of palladin concentration on the linear elasticity of the network was smaller than that of some actin bundling proteins such as fascin or scruin [18], [35], but similar to the that of filamin [39]. At higher palladin concentrations, 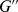 showed a shallow dip as is characteristic of viscoelastic networks, which is likely related to the rate of unbinding of palladin from actin filaments. At very high crosslinker concentrations (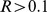), the formation of regions of dense bundle clusters interspersed with regions that have large gaps likely affected the continuity of the network, resulting in a decrease in overall stiffness of the network, as we observed. So, while palladin was efficient at creating bundled networks over a range of concentration values, there was a rapid transition to a phase where the structural integrity of the network collapsed and the network softened. This was in contrast to networks of filamin, fascin or scruin which form well-defined bundled networks with increasing network stiffness over a much larger concentration range upto (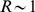).

Since many actin crosslinkers have been shown to cause actin networks to strain harden under large strains [28], [39], an open question is whether actin/palladin networks also show significant nonlinear elasticity. One technique to study nonlinear behavior is to apply a constant shear rate 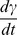 and measure the differential modulus 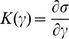 from the observed 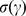. This method avoids viscous creep, which is typically non-negligible. A linear stress-strain relationship will appear flat, and any deviation from flatness will represent non-linearity. With increasing crosslinker concentration, 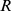, we found that actin/palladin networks exhibited limited or no strain hardening for all 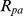 values examined (Fig. 3c). We also found that at the highest palladin concentration, 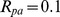, the maximum strain that the network seems to be able to accommodate before rupturing is quite large.

In general, the nonlinear mechanical response of semiflexible polymer networks may be influenced by several factors, such as the crosslinker density, unbinding kinetics, compliance of the crosslinker, actin concentration and the applied strain rate [40]. The lack of strain stiffening in palladin/actin networks may be due to a number of reasons. First, palladin may be overall a stiffer, less compliant crosslinker, unlike filamin which is a large flexible crosslinker [41]. Second, the lack of strain hardening at low 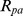 may be attributable to the low abundance of bundles, and the network being able to accommodate any applied strain (for the applied strain rate) due to the relatively rapid unbinding of palladin from filaments (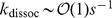), allowing for network rearrangement. Hence, most of the network may undergo remodeling and homogeneous stress redistribution, precluding any nonlinear elastic behavior, even at high strains [20], [42]. On the other hand, the extensive bundle formation at higher 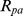 and forced crosslinker unbinding between bundles again likely results in strain accommodation, similar to observations in fascin [35], [43].

### Structure and viscoelastic properties of composite networks

**Figure 4:**
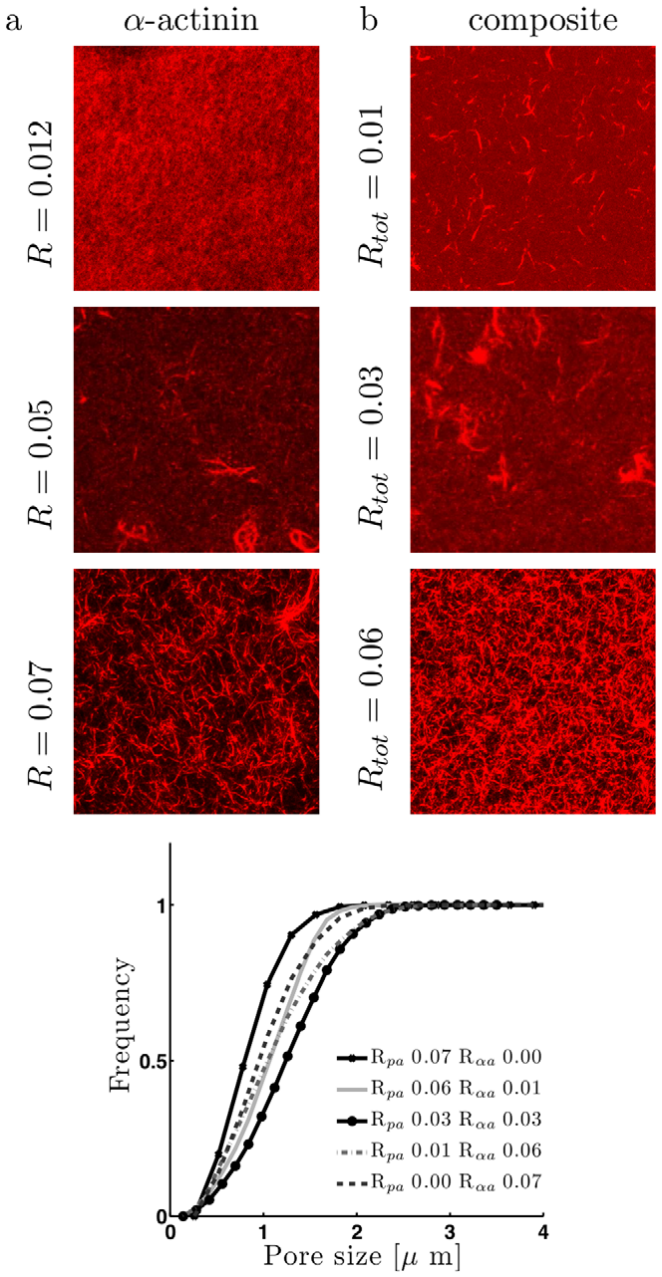
Structural properties of composite palladin/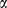-actinin networks. Maximum intensity projections of confocal stacks of actin networks formed with (a) 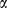-actinin and (b) mixtures of 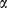-actinin and palladin (right column). Note the similarity to the projections of pure actin/palladin networks shown in Fig. 1. As the concentration of crosslinkers increases the networks transition from crosslinked actin filaments to bundled networks, and finally to clusters of bundles. (c) Pore size analysis of composite actin networks (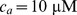). With the total crosslinker concentration held at 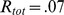, we find that addition of a small amount of palladin to 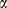-actinin/actin increases the median pore size but pure networks have smaller pore sizes than the composite networks. Note that only two significant digits of concentration ratios are shown in legend.

Given that palladin binds to 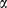-actinin with a similar affinity as to f-actin, we reasoned that composite networks of palladin/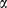-actinin/actin may exhibit distinct structural and mechanical properties compared to networks with only one type of crosslinker. In particular, we wished to examine whether the addition of either crosslinker supplements the effects of the other, i.e. whether the mutual interaction between palladin and 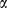-actinin leads to a synergistic or antagonistic effect on the composite networks. Confocal images of 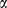-actinin and composite 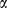-actinin/palladin actin networks are shown in Figure 4a–b. Qualitatively, the structures of networks formed by either crosslinker were very similar in terms of bundle curvature, branching, fluorescence intensity (apparent bundle thickness) and homogeneity (compare with pure palladin networks in Fig. 2). The transitions from entangled F-actin networks to bundles to clusters occurred at similar concentration ratios for all cases. We found that composite networks of 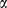-actinin and palladin also have a morphology that is very similar to singly crosslinked networks. As we increased the total crosslinker to actin ratio (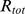), with equal amounts of 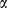-actinin and palladin in the mixture, the networks transitioned from small, isolated bundles to homogenously bundled crosslinked networks and finally to heterogenous networks with large bundle clusters at high concentrations (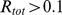.) The bundling transition appeared to be at a slightly lower concentration for the composite network compared to the actin/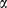-actinin network. For pure 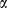-actinin (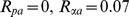), networks had median pore size slightly less than 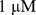 as shown in the cumulative distribution of pore-sizes, with very few large pores (Fig. 4c). Addition of small amounts of palladin (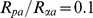) to the network shifted the pore-size distribution to the right, indicating larger pore sizes and more efficient bundling. However, higher palladin/alpha-actinin ratios resulted in a reduction of the median pore size and an overall leftward shift of the distribution. The pore size distribution for a pure palladin network was similar to that of pure alpha-actinin.

**Figure 5:**
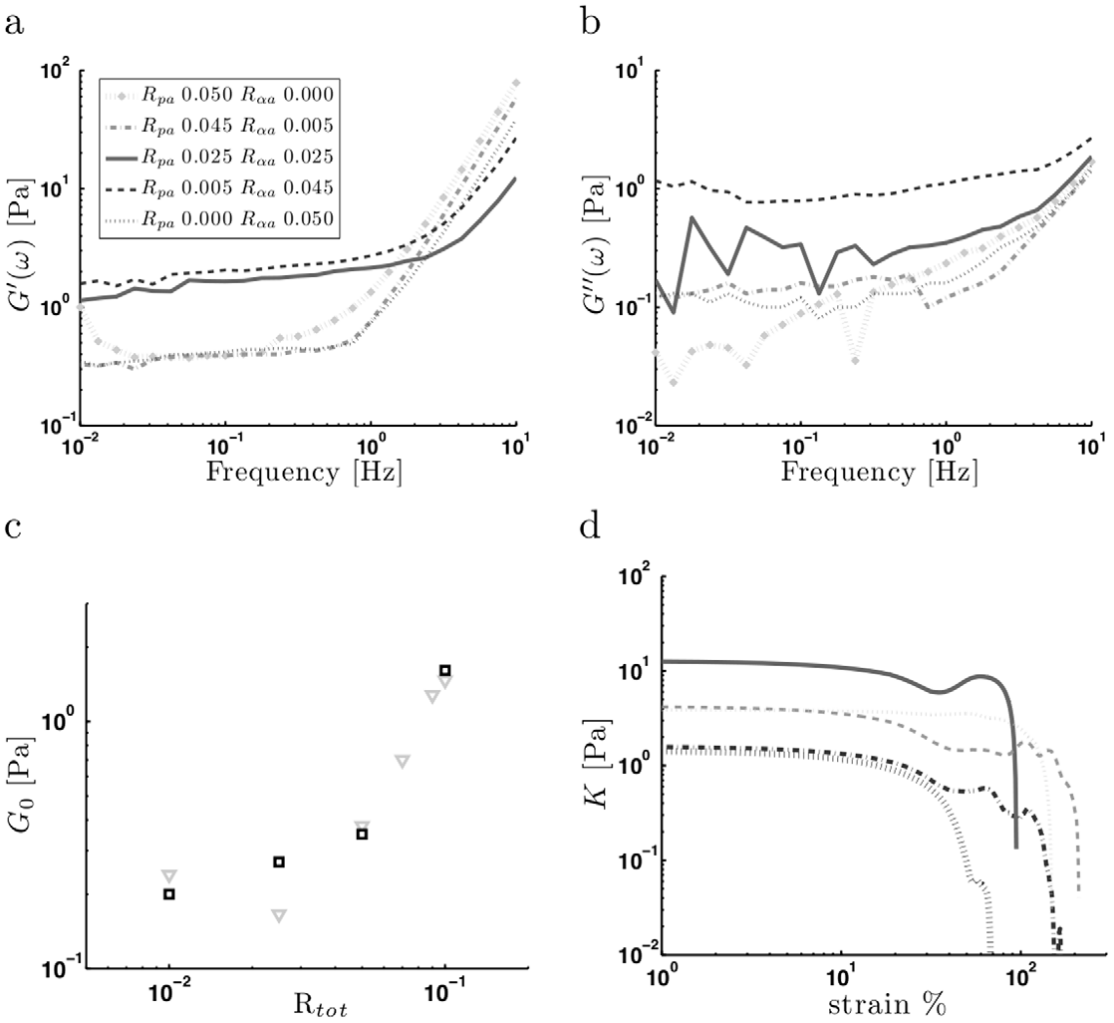
Viscoelastic properties of composite actin networks. (a) Storage moduli (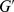), (b) loss moduli (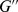) of actin networks (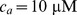) with varying concentrations of palladin and 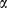-actinin, 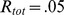. Composite networks with equal amounts of palladin and 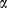-actinin are slightly stiffer than the others. (Same legend for a–b). (c) Plateau modulus, 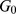 of pure palladin (gray triangles) and pure 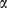-actinin (black squares) networks showing correlation between the total amount of crosslinker and stiffness, 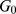. (d) Differential modulus of composite networks in (a).

We next examined the mechanical properties of composite networks to determine whether the two crosslinkers behave synergistically or independently (Fig. 5a–b). We fixed the total concentration of crosslinkers at 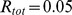 to ensure that the network was in a bundled regime. We found that the addition of palladin to pure 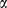-actinin networks resulted in a biphasic effect on the network stiffness (Fig. 5a). For 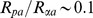, the network was considerably stiffer than for 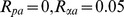. However, increasing 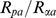 further, softened the network. The stiffness of pure palladin networks (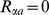) was similar to that of the pure 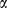-actinin networks (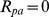). This behavior of network stiffness appeared to parallel the dependence of pore size on crosslinker ratio, 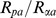 at fixed 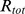 (Fig. 4c). From these data, it appears that the effect of adding small amounts of palladin to an actin/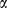-actinin network is not the same as adding a small amount of 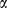-actinin to an actin/palladin network.

The mechanical properties of composite networks can be compared by studying the static stiffness of the networks, or equivalently the plateau modulus, 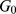, as a function of crosslinker composition. We found that 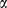-actinin and palladin networks exhibited nearly identical changes of 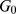 with respect to crosslinker concentration (Fig. 5c). 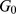 was weakly dependent on concentration for 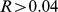 (corresponding to a low abundance of bundles in the confocal images) and increased as 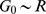 for an intermediate range of 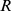 (majority of filaments were part of a crosslinked bundled network). For two independent crosslinkers, we would expect the plateau modulus, 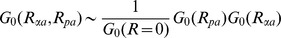 [30]. In this case, for a fixed 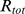, varying the ratio of the two crosslinkers would result in a monotonic change in the plateau modulus, rather than the biphasic behavior that we observed. Moreover, the independent model predicts that when two crosslinkers with similar 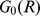 (such as palladin and 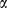-actinin) are mixed, the overall network stiffness should not depend on the exact ratio of the two crosslinkers, whereas they should for the case when 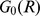 differ. However, as shown in Figure 5c, we found that the plateau moduli of composite networks was different for the same overall crosslinker ratio, depending on whether palladin or 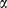-actinin was dominant. Yet the overall dependence of 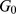 on 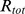 for the composite network was similar to that of either crosslinker alone. Finally, as with the case of pure palladin networks, the differential modulus showed that the addition of palladin to 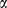-actinin did not result in strain-stiffening (Fig. 5d).

## Discussion

We have shown that palladin is an actin binding protein that crosslinks actin filaments into viscoelastic networks. Increasing palladin concentrations causes structural transitions in actin networks from a weakly crosslinked phase to a strongly bundled phase in which branched bundles span the entire network. At high concentrations, clusters of bundles form leading to a more heterogenous structure. We have also shown that palladin modulates the morphology and viscoelastic response of actin networks in a concentration-dependent manner. Actin/palladin networks stiffen with increasing palladin concentrations until a disordered cluster phase is formed and the network softens. A few other ABPs may use Ig-like domains to bind actin (myotilin, kettin), however none of these have been studied *in vitro* in terms of their ability to form networks and how they modulate network mechanics.

*In vivo* studies have shown that up-regulation of palladin leads to increased stress fiber formation and dramatic changes in cell morphology, while decreased palladin expression in cells leads to loss of f-actin and stress fibers [7]. *In vivo*, palladin participates in additional regulation of the actin cytoskeleton and the consequent mechanical properties of cells by virtue of its interactions with several actin-associated proteins, including 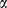-actinin. Since both palladin and 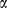-actinin are major components of stress fibers, we compared the function of palladin *in vitro* with 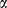-actinin and also investigated composite networks crosslinked with palladin and 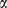-actinin. We found that the morphology and viscoelastic properties of actin/palladin networks are qualitatively very similar to that of actin/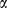-actinin networks, despite differences in their actin binding domains (Ig-like domain for palladin [8] versus calponin-homology domain containing motifs for 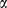-actinin [44]).

In composite networks, palladin and 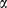-actinin did not influence actin network properties independently. Addition of low to moderate concentrations of palladin to 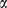-actinin networks augmented their stiffness, while addition of low concentrations of 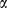-actinin to palladin networks did not alter network properties. Our results suggest that palladin and 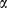-actinin do not modify the linear and nonlinear viscoelastic properties of crosslinked actin networks independently. This synergistic action might arise due to the direct interaction of palladin and 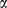-actinin. Palladin bound to 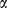-actinin might offer more actin binding sites in a smaller volume, enabling the formation of tighter bundles and facilitating bundle interconnectivity. However, at higher concentrations, palladin may play a more dominant role by displacing 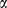-actinin from actin filaments. These studies suggest that the relative abundance of palladin and 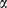-actinin might also be important in shaping cellular viscoelasticity. It remains unclear whether the distinct structural and viscoelastic properties of composite networks arise as a result of the mutual interaction between palladin and 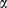-actinin or because of the difference in their actin binding domains. Studies with palladin mutants that lack 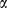-actinin and/or actin binding sites may help elucidate the specific contributions of various protein-protein interactions to the overall network mechanics. Our study highlights the importance of mutual interaction among actin crosslinkers in determining the overall structure and mechanics of actin networks.

## Materials and Methods

### Protein Isolation

Acetone powder was prepared from frozen rabbit muscle (Pel-Freeze Bologicals, Rogers AR) to purify actin for in vitro studies according to protocols approved by the NHLBI Institutional Animal Care and Use Committee. Actin was extracted by dissolving ace- tone powder in G-buffer (2 mM Tris, 0.2 mM ATP, 0.1 mM CaCl2, 0.5 mM DTT, 1 mM NaN3). The solution was centrifuged at 25000 rpm for 30 min. and then filtered through glass wool to remove the powder. Polymerization was initiated and actin was removed from solution by centrifugation. The actin was again dissolved in G-Buffer and dialyzed for 2 days. For fluorescent labeling of actin networks either AlexaFluor-488-actin (Invitrogen, Carlsbad CA) or Rhodamine-actin (Cytoskeleton, Denver, CO) was obtained. 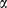-actinin was purchased from Cytoskeleton Inc. (Denver, CO). Full-length palladin (90 kD isoform) was generated using the baculovirus expression system as detailed in Dixon *et al.* [8]. The target gene was amplified via PCR. This portion of DNA was added to a plasmid and transfected into *E. coli*, which in turn was used to infect insect cells. After enough *E. coli* expressing palladin have been produced, the protein was isolated with immobilized nickel columns.

### Actin polymerization

Unlabeled and labeled actin monomers, crosslinkers (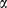-actin and/or palladin) and G-buffer were mixed on ice to obtain the desired ratios of proteins. The final concentration of actin monomers ranged from 1 to 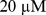 with 10% of the actin labeled with either Rhodamine or Alexa-Fluor-488. The concentration of ABPs varied from 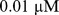 to 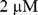. After polymerization was initiated by adding 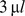 of 10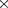 polymerization buffer (1 M KCl, 20 mM MgCl2, 20 mM tris-HCl and 10 mM ATP), the samples were immediately pipetted into wells for imaging, or onto the bottom plate of the rheometer. Wells were made using 0.5 mm thick silicone spacers with circular holes (diameter 9 mm) placed on a No.1 coverglass and the top was sealed with a microscope slide. The overall volume of each well was 30 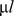. After pipetting on to the rheometer bottom plate, the top cone was immediately lowered into the sample and surrounded by a solvent trap made using a metal surround with a sponge soaked in water.

### Imaging

Images were gathered on a Zeiss LSM 510 or LSM 710 confocal microscope using a 63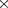 oil immersion objective. A 543 nm HeNe laser line was used to excite Rhodamine labeled actin, and a 488 nm Argon line was used for exciting the Alexa488 labeled actin. The images were 512
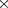
512 pixels (73.1
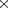
73.1 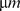) digitized at 8 bits per pixel. All data presented was corrected for any variation in the image acquisition settings. The slice thickness for confocal images varied from 0.24 to 1 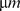 between stacks, but each stack used a consistent spacing.

### Image Analysis

Pore size distributions were calculated from confocal images. There is no generally accepted definition of pore size distribution, and some methods, such as peak to peak distance measures [18], can give biased estimates (e.g. in a directional network). We used a robust method that uses volumes of covering spheres to calculate pore sizes [45]. In this method, the confocal stack is first converted to a 3D binary image, with bundle voxels labeled as 1 and fluid voxels (background or empty space) labeled as 0. A distance map is computed for every fluid voxel representing the distance from each fluid voxel to the nearest bundle or 1 voxel. Then, starting from the voxels with the smallest distance value, covering spheres with centers on that voxel were generated. The size of the sphere was chosen such that it is the largest possible sphere that did not cover a bundle or 1 voxel. All voxels within the sphere are given the same value as its center voxel, unless its distance map value was greater. This ensures that large spheres covered smaller ones. In the end, each background voxel is marked with the size of the largest sphere which covered it. These values are binned to produce the pore size distribution. In our studies, networks that were homogeneous throughout the confocal stacks were chosen for pore size analysis. Pores that reached the edge of the confocal stack were ignored, as an abundance of such pores causes the pore size analysis of these networks to fail.

### Rheometry

Bulk physical properties of the networks were measured with a custom Anton Paar MCR-301 stress-controlled bulk rheometer. The upper portion is cone shaped so that the strain is constant throughout the sample. A solvent trap was used to surround the sample and cone head of the rheometer in order to minimize evaporation. The sample (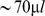 volume) rested on a plate at the base of the rheometer, and a 40 mm diameter, 1 degree cone was lowered into it from above with a 50 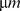 spacing. It was assumed that the sample does not slip on either surface. To characterize the linear viscoelastic response, small amplitude, oscillatory shear strain, 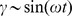, was applied and the resultant oscillatory stress, 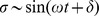, was measured, where 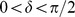 is the phase shift of the measured stress. The in-phase component of the stress response determines the shear elastic modulus, 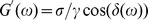 which is a measure of how mechanical energy is stored in the material. The out-of-phase response measures the viscous loss modulus, 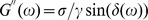 which is a measure of how mechanical energy is dissipated in the material. Yield tests are carried out at a continuous strain of 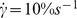. The force required to drive this deformation is recorded at 0.1 s intervals. The differential modulus (defined as 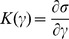) is computed numerically from this data.
